# Protective Effect of Salidroside Against Diabetic Kidney Disease Through Inhibiting BIM-Mediated Apoptosis of Proximal Renal Tubular Cells in Rats

**DOI:** 10.3389/fphar.2018.01433

**Published:** 2018-12-04

**Authors:** Congcong Guo, Yun Li, Rui Zhang, Yaqin Zhang, Junyu Zhao, Jinming Yao, Jie Sun, Jianjun Dong, Lin Liao

**Affiliations:** ^1^First Clinical Medical College, Shandong University of Traditional Chinese Medicine, Jinan, China; ^2^Division of Endocrinology, Department of Internal Medicine, Shandong Provincial QianFoShan Hospital, Shandong University, Jinan, China; ^3^Department of General Health Care II, Shandong Provincial Hospital Affiliated to Shandong University, Jinan, China; ^4^Division of Endocrinology, Department of Internal Medicine, Qilu Hospital of Shandong University, Jinan, China

**Keywords:** diabetic kidney disease, salidroside, apoptosis, BIM protein, proximal renal tubular epithelial cell

## Abstract

**Background:** Accumulating evidences indicate that the apoptosis of proximal tubular epithelial cells (PTECs) play a vital role in the progression of the diabetic kidney disease (DKD). This study aimed to explore the therapeutic potential of salidroside (SAL) in DKD and its underlying mechanism in anti-apoptosis of PTECs.

**Methods:** Twenty-eight male Wistar rats were allocated into four groups: sham-operated, uninephrectomy (unx), diabetes with uninephrectomy (DKD) and DKD treated with SAL (DKD + SAL). SAL (70 mg/kg) was gavage administered for 8 weeks. 24-h albuminuria and serum creatinine (SCr), blood urea nitrogen (BUN), renal histological changes were examined. The silico analysis was used to identify the main therapeutic targets and pathways of SAL involved in DKD treatment. Apoptosis was determined by TUNEL and Annexin V-FITC/PI double staining *in vivo* and *in vitro*, respectively. The expression of BIM, BAX, and cleaved caspase-3 were evaluated by western blot and immunostaining.

**Results:** Treatment with SAL significantly attenuated diabetic kidney injury via inhibiting 24-h albuminuria, SCr, BUN, glomerular mesangial dilatation and tubular injury in DKD rats. The silico analysis identified the intrinsic apoptotic pathway as an important pathway responsible for the nephroprotective properties of SAL. Our data validated that SAL effectively inhibited the apoptosis of PTECs induced by high-glucose (HG), both *in vitro* and *in vivo*. Silence of BIM by shRNA in HK-2 cells prevented HG-induced apoptosis. The up-regulated BIM and its downstream targets (BAX and cleaved caspase-3) were also inhibited by SAL.

**Conclusion:** In summary, SAL significantly relieved DKD. And the possible mechanisms might be partially attributed to inhibiting apoptosis of proximal renal tubular cells. The apoptotic protein BIM could be an important target of SAL in this process.

## Introduction

Diabetic kidney disease (DKD) has become the leading cause of chronic renal failure around the world (Afkarian et al., 2016; Zhang L. et al., 2016). Most researchers agree that initial lesions in DKD affect the glomerular compartment, especially podocytes (Lin et al., 2015). However, the progression of the disease reflects the stronger correlation with the impairment of tubules, especially proximal tubule (Gilbert and Cooper, 1999; Schnaper, 2017). Recent studies also indicated that the proximal tubulopathy might occur earlier than glomerulopathy and in turn contribute to the irreversible glomerular pathology (Hasegawa et al., 2016). As we know, the proximal tubular regions generate a large amount of energy to sustain the hyper-reabsorption function, which is accompanied by glomerular hyperfiltration in early DKD. The high energy requirement and aerobic metabolism in the proximal tubular epithelial cells (PTECs) render it particularly susceptible to injury (Gilbert, 2017). Apoptosis, one of the characteristic morphologic changes in tubules, has been recognized as a major cause of renal fibrosis. The inhibition of apoptosis might be helpful in relief of DKD (Lau et al., 2012; Wang et al., 2016).

Salidroside (SAL), one of the main active constituents isolated from *Rhodiola rosea*, possesses several pharmacological activities, including anti-hypoxic (Li et al., 2016), anti-inflammatory (Chang et al., 2015), and anti-fibrotic effects (Zhang et al., 2014). The role of SAL in prevention of apoptosis has been reported in multiple cells culture, such as pheochromocytoma (PC12) cells (Hu et al., 2016), SH-SY5Y neuroblastoma cells (Wang C.Y. et al., 2018) and cardiomyocytes (Zhu L. et al., 2015). However, its effects on the apoptosis of PTECs induced by diabetes have not been evaluated. Therefore, the present study is designed to investigate the effects of SAL on DKD prevention and then to explore the underlying mechanism in anti-apoptosis of PTECs *in vivo* and *in vitro*. We found that SAL indeed had the potential to improve DKD, which was associated with a significant inhibition of BIM-mediated PTECs apoptosis.

## Materials and Methods

### Animal Models and Treatment

Male Wistar rats with initial body weights of 200–250 g were purchased from Beijing HFK Bio-Technology Co., Ltd. All animals were housed in a room with constant temperature and a 12:12-h light–dark cycle. They were allowed free access to a standard diet and tap water. The rats were allowed to acclimatize for 1 week before the experiment began. Sham control rats underwent a sham operation consisting of an incision in the skin and muscle in the right renal region and manipulation of the renal pedicles, without damage to the kidney. To accelerate the DKD development, the other rats were subjected to right uninephrectomy at 8 weeks of age (Huang et al., 2009). One week after uninephrectomy, diabetes was induced by a single intraperitoneal injection of streptozocin (STZ; Sigma-Aldrich, St. Louis, MO, United States), diluted in a citrate buffer (0.1 mol/L, pH 4.0), at a 45 mg/kg dose. Finally, 16 rats developed hyperglycemia, with blood glucose levels over 16.7 mmol/L, at 72 h after STZ injection. After confirmation of hyperglycemia in the diabetic rats, all animals were randomized into four groups: (1) sham (*n* = 6), (2) uninephrectomy (unx; *n* = 6), (3) diabetes with uninephrectomy (DKD; *n* = 8), and (4) diabetes with uninephrectomy treated with SAL (DKD + SAL; *n* = 8). The rats in the sham and unx groups, which were not induced with STZ, were used as non-diabetic controls. SAL (purity > 98%; National Institutes for Food and Drug Control, Beijing, China) was administered daily by gavage at a dose of 70 mg/kg body weight for 8 weeks in the SAL-treated rats (Zheng et al., 2015), while the other groups received the vehicle control without SAL. Blood glucose levels were monitored at least weekly in all diabetic rats by tail-vein blood sampling. After 8 weeks, one rat died in the DKD group. The rats were housed individually in metabolic cages for urine collection. Within 1–2 days after the last urine collection, the animals were sacrificed. Blood samples were obtained, and the left kidney was immediately removed. Part of the kidney tissue was fixed in 4% paraformaldehyde, while the remaining tissue was stored at -80°C. The study was conducted in accordance with the Guiding Principles for the Care and Use of Laboratory Animals of China, and the protocol was approved by the Ethics Committee of Shandong Provincial QianFoShan Hospital, China.

### Biochemical Analysis

Renal function was assessed by measuring the kidney index, 24-h urine protein and albumin, blood urea nitrogen (BUN), and serum creatinine (SCr) of the rats. The kidney index (in mg/g) was calculated as a ratio of the left kidney’s weight to the body weight (K/W). Urine protein was assessed by the Bradford method, while urine albumin was measured using an enzyme-linked immunosorbent assay kit (CUSABIO Engineering Co., Wuhan, China). Plasma biochemical parameters were measured using an automatic biochemical analyzer (Chemray 240; Rayto, Institute of Biotechnology, Shenzhen, China).

### Histological Observation

The removed kidney tissues were fixed in 4% paraformaldehyde and embedded in paraffin. Paraffin sections (3–4 mm) were stained with periodic acid-Schiff (PAS), periodic acid silver methenamine (PASM) and Masson’s trichrome. The sections were examined with light microscopy by two experienced pathologists. The index of mesangial expansion represented the percentage of PAS-positive area in the glomerulus. It was scored by a quantitative estimation of the width of mesangial zones at 40× power for 20 cortical fields. Injury to tubules was assessed by determining the percentage of affected tubules per 10 fields (magnification ×200) (Zhao et al., 2014). The scoring system was on a scale from 0 to 5 grades (0 = 0%, 1 = 5%, 2 = 5–10%, 3 = 10–20%, 4 = 20–30%, 5 = >30%) according to the following criteria: tubular dilation, tubular atrophy, vacuoles formation, and extracellular matrix accumulation (interstitial volume).

### Electron Microscopy

Cortical kidney tissue was cut into 1 mm^3^ cubes for standard Electron Microscopy processing. Photographs were taken with transmission electron microscope (JEM-1200EX, Japan). Five random photographs with a final magnification of 15,000× were taken from each section.

### Identified Targets of SAL in DKD Treatment

The genes related to DKD were selected from six existing databases: (1) the DrugBank database (Wishart et al., 2008), (2) the Comparative Toxico genomics database (CTD) (Davis et al., 2018), (3) the Online Mendelian Inheritance in Man (OMIM) (Amberger and Hamosh, 2017), (4) the Therapeutic Target database (TTD) (Liu et al., 2011), (5) the Kyoto Encyclopedia of Genes and Genomes (KEGG) Pathway database (Kanehisa et al., 2017), and (6) the Genetic Association database (GAD) (Becker et al., 2004). Based on the inference score computed by the CTD database, we extracted the targets scored above 60.

The targets of SAL were extracted from the Herbal Ingredients’ Targets (HIT) database (Ye et al., 2011), the Swiss Target Prediction database (Gfeller et al., 2014), the STITCH 5.0 database (Szklarczyk et al., 2016) and the ChemMapper database (Gong et al., 2013). A Canonical SMILES (C1=CC(=CC=C1CCOC2C(C(C(C(O2)CO)O)O)O)O) was recorded for SAL (PubChem CID: 159278) from the PubChem database and separately uploaded to the servers. In the ChemMapper database, if the two-dimensional similarity of a molecule was above 0.6, the targets were predicted as the targets of SAL. Noteworthy, all the species of the targets was *Homo sapiens*. Then, to better dissect the role of SAL in DKD treatment, those targets were mapped to DKD-related genes to obtain the candidate targets of SAL.

### Protein–Protein Interaction Network Construction and Pathway Analysis

To normalize the gene information, inconsistent ID types were converted to UniProt ID accession numbers. The associated genes of the candidate targets were obtained from the String database (Jensen et al., 2009). Among them, only the protein with a interaction score above 0.9 were carried on for constructing the protein–protein interaction (PPI) network using Cytoscape version 3.2.1 (Shannon et al., 2003). The topological property of each node in the network was calculated by Network Analyzer (Batt et al., 2012). A node with a degree value two times greater than the average degree (5.719) was considered to an important node (Zhang Y. et al., 2016). Cytoscape plugin Reactome (Vastrik et al., 2007) was employed to perform pathway analysis of the key nodes of SAL in DKD treatment. The *P*-value < 0.01, FDR < 0.01 were set as the cut-off criteria. Furthermore, the top 10 signaling pathways correspond to the criteria, were displayed as bubble charts by the E Chart online analysis platform a histogram.

### Cell Culture and Transfection

HK-2 cells, a proximal tubule epithelial cell line, were purchased from PriCells (Wuhan, China) and cultured as described previously (Cai et al., 2017). The cells were incubated in media containing 5.5 mM D-glucose + 24.5 mM D-mannitol (MG), 30 mM D-glucose (HG), or 30 mM D-glucose + 100 μM SAL (HG + SAL). D-glucose and D-mannitol were purchased from Sigma-Aldrich Canada Co. (Oakville, ON, Canada).

The short hairpin RNA (shRNA) targeting human *BIM* (5′-CGGAGACGAGTTTAACGCTTA-3′) was designed, synthesized, and cloned into the hU6-MCS-ubiquitin-EG FP-IRES-puromycin vector (*BIM*-shRNA) by the GeneChem Corporation (Shanghai, China). The hU6-MCS-ubiquitin-EGFP-IRES-puromycin vector (shNC) was used as a negative control. After confirming successful transfection, HK-2 cells were cultured in high glucose medium for 48 h and then harvested for RNA or protein extraction.

### Cell Counting Kit-8 Assay

HK-2 cells were plated on 96-well plates at a density of 3 × 10^4^ cells/cm^2^ in RPMI 1640 medium. Firstly, cells were incubated in 30 mM D-glucose medium for 24, 48, and 72 h, respectively. The cell viability was assessed by the Cell Counting Kit-8 assay (Dojindo Molecular Technologies, Inc., Kumamoto, Japan) according to the manufacturer’s protocol. Secondly, the cells were incubated in 5.5 mM D-glucose + 24.5 mM D-mannitol (MG), 30 mM D-glucose (HG), or 30 mM D-glucose added to SAL at various concentrations (0.1, 1, 10, 100, 1,000, and 10,000 μM). After 72 h, cell viability was assessed by the Cell Counting Kit-8 assay. Absorbance of the samples was measured at a wavelength of 450 nm.

### TUNEL Assay

The apoptotic cells were determined by the terminal deoxynucleotidyl transferase-mediated dUDP nick-end labeling (TUNEL) technique using an *in situ* cell death detection kit (Roche). Paraffin sections of the kidney were cut at 3 μm and deparaffinized for staining. The paraffin sections were assessed by the TUNEL assay according to the manufacturer’s recommendations. At least 10 renal cortex fields per slide and six slides per group were scored for apoptotic nuclei. The apoptotic cells from tubular microscopic fields (magnification ×400) were counted. Data were expressed as positive cell count, which is the mean of cells positive for apoptotic cell microscopic field (An et al., 2013; Xiao et al., 2014).

HK-2 cells were seeded on sterile glass coverslips in a 6-well plate and stimulated with 5.5 mM D-glucose + 24.5 mM D-mannitol (MG), 30 mM D-glucose (HG), or 30 mM D-glucose + 100 μM SAL (HG + SAL) for 48 h. In addition, HK-2 cells transfected with shNC and *BIM* shRNA were incubated in high glucose for 48 h. The HK-2 cells were assessed by the TUNEL assay according to the manufacturer’s recommendations. TUNEL-positive cells were counted under the light microscope by two independent pathologists. The rate of apoptosis in HK-2 cell was calculating by dividing the number of TUNEL positive cells to a population of 100 counted cells per microscopic field (magnification ×40 or ×100).

### Annexin V-FITC/Propidium Iodide Assay

An Annexin V–fluorescein isothiocyanate (FITC) apoptosis detection kit (Neobioscience, Inc., Shenzhen, China) was used to assess the apoptosis. HK-2 cells were seeded in 6-well plates. The adhered cells were exposed to different media for 24 h or 72 h and then detached with trypsin. Subsequently, the cells were resuspended in a binding buffer (195 mL) and stained with Annexin V–FITC (5 μL) and propidium iodide (PI; 10 μL) at room temperature for 10 min in the dark. Apoptotic cells were determined using a flow cytometer (BD FACSAria^TM^ II cell sorter). Annexin V–FITC-positive and PI-negative cells were considered apoptosis.

### Quantitative Real-Time Polymerase Chain Reaction

HK-2 cells were stimulated with different media for 48 h, and then RNA isolation and cDNA synthesis were performed as previously described (Cai et al., 2017). Gene expression was measured by real-time polymerase chain reaction (PCR) using 0.2 μM gene-specific primers and the UltraSYBR One Step RT-qPCR kit (low ROX) (Takara Bio, Inc., Otsu, Japan) in a total volume of 20 μL. PCR was performed using an Applied Biosystems ViiA 7 sequence detection system. Relative expression levels of the *BIM* gene were determined by the ΔΔCt method, whereby the target gene expression was normalized to that of β-actin as an endogenous reference and then compared to the control. Each experiment was repeated three times. The primer sequences were as follows: *BIM* forward (5′-ATTACCAAGCAGCCGAAGAC-3′) and reverse (5′-TCCGCAAAGAACCTGTCAAT-3′); and β-actin forward (5′-TGACGTGGACATCCGCAAAG-3′) and reverse (5′-CTGGAAGGTGGACAGCGAGGT-3′).

### Western Blotting

Proteins were isolated from cultured HK-2 cells and animal renal cortices. Cell lysates (30–100 μg) were analyzed by immunoblotting as described previously (Zhang et al., 2017). The antibodies used were anti-BIM (monoclonal) from Abcam (1:500), anti-BAX (1:1,000, polyclonal) and anti-cleaved caspase-3 (1:500, polyclonal) from Affinity, and anti-β-actin from ZsBio (Beijing, China). Quantification was performed by measuring the signal intensity using the ImageJ software (National Institutes of Health, Bethesda, MD, United States).

### Immunostaining

For immunohistochemistry, the antibodies used in this study included a B-cell lymphoma 2 (BCL-2)-interacting mediator of cell death (BIM) rabbit antibody (1:100; Abcam, Cambridge, MA, United States), neutrophil gelatinase-associated lipocalin (NGAL) rabbit antibody (1:100, Abcam, Cambridge, MA, United States); a BCL-2-associated X (BAX) rabbit antibody (1:100; Affinity, Chicago, IL, United States), and a BCL-2 rabbit antibody (1:100; Affinity). Slides were incubated with the antibodies for 12 h at 4°C. Negative controls were incubated with phosphate-buffered saline (PBS). The bound antibodies were detected with horseradish peroxidase-conjugated anti-rabbit IgG and diaminobenzidine. Finally, the slides were counterstained with hematoxylin. All images, acquired from randomly selected microscopic fields, were measured using Image-Pro Plus 6.0 (Media Cybernetics, Silver Spring, MD, United States) as described previously (Tervaert et al., 2010).

For immunofluorescence, HK-2 cells were seeded on sterile glass coverslips in a 6-well plate and stimulated with different media for 48 h. The slides were fixed for 10 min with 4% paraformaldehyde, followed by permeabilization with 0.05% Triton X-100 in PBS for 15 min. Thereafter, the slides were incubated with the BIM rabbit antibody (1:150; Abcam) at 4°C for 24 h, followed by incubation with a secondary antibody (1:200 in PBS) for 1 h at room temperature in the dark. Finally, the slides were stained with diphenyleneiodonium. Images were examined under a fluorescence microscope (Olympus FSX100).

### Statistical Analysis

Data were presented as means ± standard error of the mean. SPSS 21.0 software was used for statistical analysis. Student’s *t*-test was used to assess significance for data between two groups. One-way ANOVA and two-way ANOVA test with subsequent *post hoc* Tukey’s test were used for multiple comparisons. Kruskal–Wallis test was used for non-parametric data comparison. *P* < 0.05 was considered indicative of statistical significance.

## Results

### Salidroside Decreased Urine Protein and Improved Biochemical Parameters in DKD Rats

**FIGURE 1:**
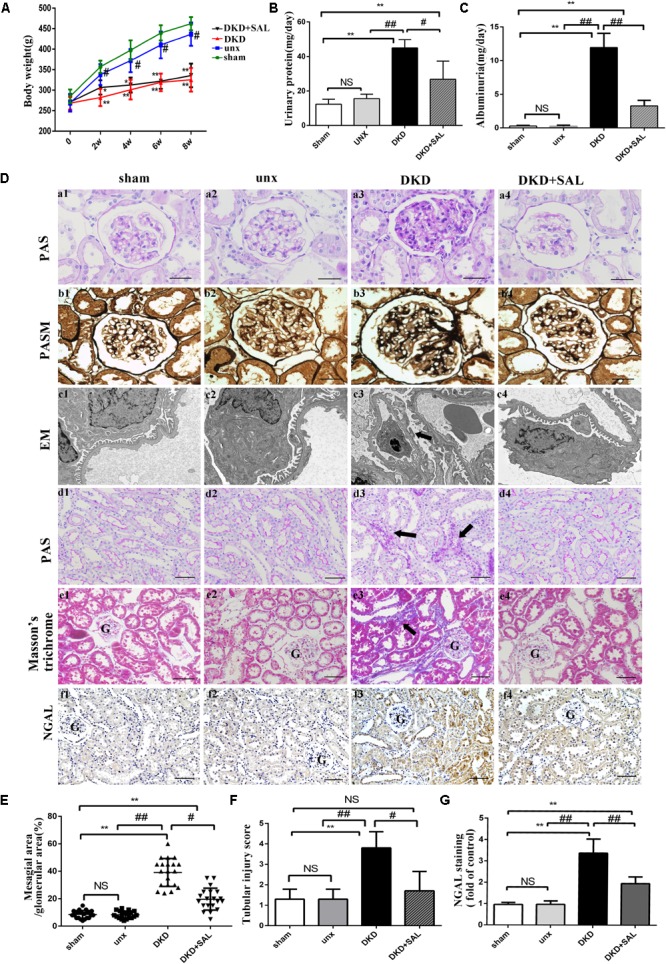
Salidroside decreased urinary albumin and attenuated renal histological injury in diabetic rats. **(A)** Body weight were assessed in sham group, uninephrectomy (unx) group, diabetes with uninephrectomy (DKD) group, and diabetes with uninephrectomy treated with salidroside (DKD + SAL) group. DKD group and DKD + SAL group showed an obvious reduction in body weight over the 8-week study. **(B,C)** Urinary protein and albuminuria. DKD group showed increased urinary protein and albuminuria, and these parameters were reduced by SAL treatment. **(D) (a1–a4, b1–b4)** Representative micrographs of periodic acid Schiff (PAS)-stained glomerular and periodic acid silver methenamine (PASM)-stained glomerular, magnification: ×400, scale bar = 30 μm. **(c1–c4)** Morphology change in the podocyte foot process (transmission electron microscopy, ×15,000). The fusion and effacement of foot processes were observed in DKD group compared to sham group and unx group, which was less serious in SAL-treated rats. **(d1–d4, e1–e4)** Representative micrographs of periodic acid Schiff (PAS)-stained tubule and Masson’s trichrome-stained sections, magnification: ×200, scale bar = 50 μm, G, glomerulus. **(f1–f4)** Tissue expression of neutrophil gelatinase-associated lipocalin (NGAL) in kidneys, magnification: ×200, scale bar = 50 μm, G, glomerulus. **(E)** The degree of mesangial matrix expansion was scored as the percentage of PAS-positive area in the glomerulus. DKD group showed increased mesangial expansion, and SAL tended to reduce mesangial expansion. **(F)** The score of tubular injury. Tubular injury was evaluated by blinded scoring of injury tubule in PAS stained section at 20× power-field. **(G)** Semiquantitative analysis of NGAL expressed in kidney. The expression of NGAL was significantly upregulated in DKD group, both in proximal and distal convoluted tubules, which were largely inhibited by treatment with SAL. Values are expressed as mean ± SE, *n* = (6–8), ^∗^*P* < 0.05, ^∗∗^*P* < 0.01, vs. sham. ^#^*P* < 0.05, ^##^*P* < 0.01, vs. DKD.

There are no significant differences in body weights among groups at baseline. However, after STZ injection, diabetic rats showed an obvious reduction in body weight over the 8-week study period (Figure 1A). And there were significant increases in 24-h proteinuria and albuminuria in the DKD group at the end of week 8. SAL treatment (70 mg/kg) had no obvious effect on body weight but significantly reduced 24-h proteinuria (*P* < 0.05 vs. DKD group) and albuminuria (approximately 66.7%, *P* < 0.01 vs. DKD group, Figures 1B,C).

As expected, the results summarized in Table 1 showed that DKD rats exhibited a severe polyuria (198.12 ± 18.56 mL/24 h) when compared to non-DKD rats (10.56 ± 3.31 and 9.67 ± 1.43 mL/24 h), and this parameter was reduced by approximately 16.7% in DKD rats treated with SAL. Kidney weight/body weight ratio was 5.05 ± 0.44 mg/g in uninephrectomy rats and was significantly increased in DKD rats (∼2-fold). However, treatment with SAL failed to reverse the increase. In addition, DKD rats exhibited a significant increase in SCr (∼2.3-fold) and BUN (∼2.4-fold), when compared to non-DKD rats. SAL tended to reduce the levels of SCr (*P* < 0.05 vs. DKD group) and BUN (*P* < 0.01 vs. DKD group). These results suggest that SAL could decrease urine protein and be helpful in relief of DKD.

**Table 1:** Changes in biochemical parameters in each group.

	Sham (*n* = 6)	unx (*n* = 6)	DKD (*n* = 7)	DKD + SAL (*n* = 8)
Body weight (g)	462.17 ± 16.10	463.50 ± 28.33^#^	325.25 ± 28.55^∗∗^	325.25 ± 28.73^∗∗^
24 h urine volume (mL/day)	10.56 ± 3.31	9.67 ± 1.43^##^	198.12 ± 18.56^∗∗^	165.60 ± 18.67^∗∗#^
Left kidney weight (g)	1.74 ± 0.33	2.21 ± 0.26^#^	3.32 ± 0.32^∗∗^	3.02 ± 0.13^∗∗^
K/W (mg/g)	3.76 ± 0.61	5.05 ± 0.44^#^	10.22 ± 0.83^∗∗^	9.43 ± 0.94^∗∗^
BUN (mmol/L)	7.01 ± 0.99	6.63 ± 0.92	15.81 ± 0.22^∗∗^	12.28 ± 1.71^∗∗#^
SCr (μmol/L)	35.81 ± 3.48	35.79 ± 1.82^##^	80.97 ± 15.50^∗∗^	67.76 ± 12.53^∗∗#^

*BUN, blood urea nitrogen; K/W, kidney weight/body weight ratio; SCr, serum creatinine. Eight weeks after salidroside treatment, biochemical parameters of sham group, uninephrectomy (unx) group, diabetes with uninephrectomy (DKD) group, and diabetes with uninephrectomy treated with salidroside (DKD + SAL) group. Values are expressed as mean ± SE, n = (6–8), ^∗∗^P < 0.01, vs. sham. ^#^P < 0.05, ^##^P < 0.01, vs. DKD.*

### Salidroside Attenuated Renal Histological Injury in DKD Rats

Morphologically, representative images of histopathological slides were demonstrated in Figure 1D. There were no obvious differences in histological characteristics between the sham group and unx group. In contrast, the pathology of untreated DKD rats were characterized by mesangial matrix expansion (Figures 1D, a1–a4), glomerular hypertrophy (Figures 1D, b1–b4), the fusion and effacement of foot processes (Figures 1D, c1–c2), vacuolar formation of renal tubules (Figures 1D, d1–d4), and extracellular matrix accumulation (Figures 1D, e1–e4), which were less serious in SAL-treated rats. Specifically, treatment with SAL for 8 weeks inhibited glomerular mesangial matrix expansion (approximately 50%, *P* < 0.05, vs. DKD group) (Figure 1E). In the present study, we also made a quantitative analysis of renal tubular injury. As shown in Figure 1F, SAL treatment significantly decreased the tubular injury induced by diabetes (*P* < 0.05, vs. DKD group). Neutrophil gelatinase-associated lipocalin (NGAL) is supposed to be a marker of active renal tubular injury (Kaul et al., 2018). The expression of NGAL in tubule was independently associated with GFR decline slope (Hwang et al., 2017), which added strong evidence to the hypothesis that tubular injury plays a critical role in the progression of DKD. In the present study, immunohistochemistry assays showed a substantial increase of NGAL in the renal tubule of DKD group, while SAL treatment significant decreased the expression (*P* < 0.01, Figures 1D, f1–f4, 1G). The results provided evidence for the protective effects of SAL on diabetic kidney injury.

### Identified Targets of Salidroside in DKD Treatment

Based on the above results, we employed *in silico* analysis to excavate the therapeutic targets of SAL against DKD based on the existing databases (Wang T. et al., 2018) (Supplementary File S1, Websites). After deleting repeated targets, we collected 259 DKD-related genes from six databases (Supplementary Table S1) and 61 SAL-related targets from four databases (Supplementary Table S2). Then, the 61 targets were mapped to the DKD-related targets to obtain shared targets. Consequently, 15 targets were overlapped, including *MMP1*, ***CASP3*** *AKT1, HIF1A, IL10, MMP9, PTGS2*, ***BCL2, BAX*,** *MMP2, PTGS1, SLC6A2, PRKCA, LGALS3, BCHE*, which were predicted as the candidate targets for SAL in treatment DKD.

### Protein–Protein Interaction (PPI) Network Construction and Pathway Analysis

**FIGURE 2:**
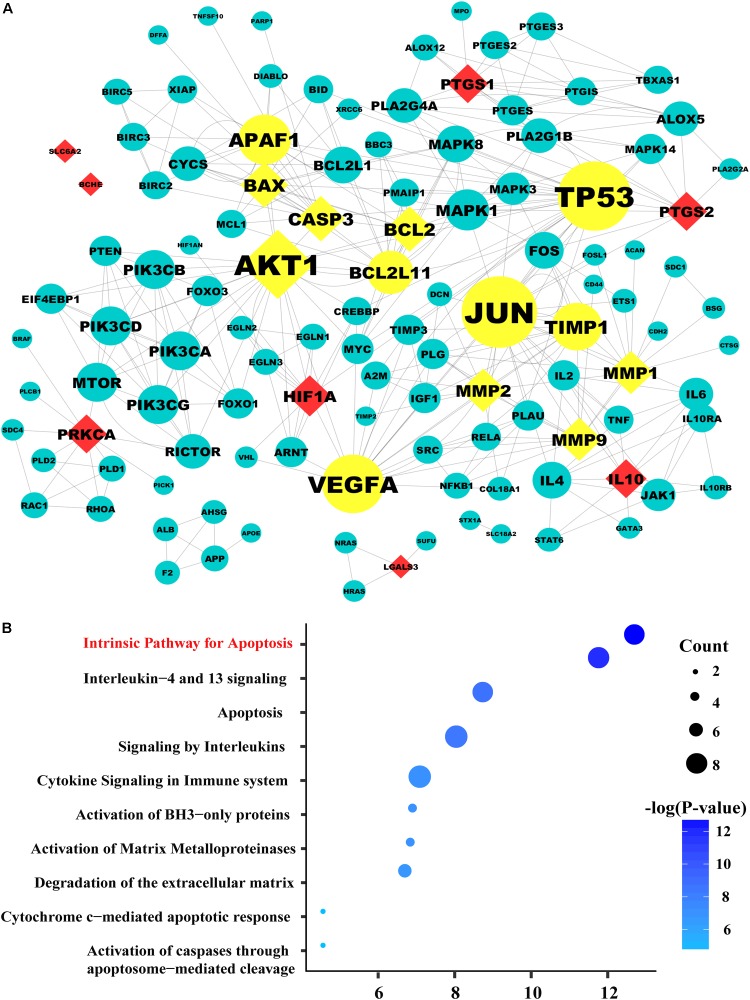
The protein–protein network and pathway analysis. **(A)** The diamond nodes represent the 15 candidate targets of SAL in treatment diabetic kidney disease (DKD), and the circular nodes represent the associated proteins of the targets. The yellow nodes represent the important genes in the network, with a degree two times larger than the average degree (5.719). The sizes of the nodes are illustrated from big to small in descending order of degree values. **(B)** Pathway enrichment of the important genes. In the bubble graph, the sizes of the bubbles are illustrated from big to small in descending order of the number of the important targets involved in the pathways. The bubble color represents the –log(*P*-value). The higher the –log(*P*-value), the deeper the bubbles color. And the intrinsic apoptosis signaling pathway exhibited the highest –log(*P*-value) value, suggesting that salidroside may exert its therapeutic effects on diabetic kidney disease by regulating the pathways related to apoptosis.

System biology studies have shown that proteins and proteins are interconnected. In order to explore the mechanism of drug treatment systematically, not only the candidate targets should be screened from the database, but also the targets associated with them. PPI network has become an important tool for screening indirect targets and evaluate the role of the targets in complex diseases (Wu et al., 2014; Sneha et al., 2018). In the present study, 101 genes associated with the 15 candidate targets were acquired from the String database, which is an efficient and accurate platform that can be used for the investigation of the PPI. The PPI network of SAL against DKD was consisted of the 116 genes through 344 interactions with an average degree of 5.719 (Figure 2A). In order to elucidate the role of the 116 targets in the PPI network of SAL in treatment DKD, the topological property of each node in the network was calculated by Network Analyzer. Generally, nodes with large degree value were considered as the important nodes of the network. As shown in Figure 2A, the 13 nodes with yellow color were considered as the important targets responsible for the nephroprotective effect of SAL, including seven candidate targets with diamond shape (***BCL2, CASP3, BAX***, *MMP2, MMP9, MMP1, AKT1*) and their related-genes (*BCL2L11* (protein name, BIM), *TP53, APAF1, TIMP1, VEGFA, JUN*) with circle shape. These genes were enriched mainly in the signaling pathway related to apoptosis, inflammation and extracellular matrix (ECM) degradation, suggesting that SAL might protect against DKD through inhibiting cell apoptosis, inflammation and fibrosis (Figure 2B).

Base on the pathway analysis, we hypothesized that the intrinsic apoptosis pathway may be an important pathway of SAL in treatment DKD. Indeed, the pathogenesis of DKD is complicated, various cells, such as podocytes, mesangial cells, tubular cells, and fibroblasts, all participate in the process. It is now increasingly recognized that the apoptosis of proximal tubular cells has an important role in the pathogenesis and progression of DKD (Gilbert, 2017; Li et al., 2017). Thus, we chose the proximal renal tubular cells as the objects to verify the anti-apoptotic activity of SAL. We validated it *in vitro* and *in vivo*.

### Salidroside Inhibited Tubular Cell Apoptosis *in vivo* and *in vitro*

**FIGURE 3:**
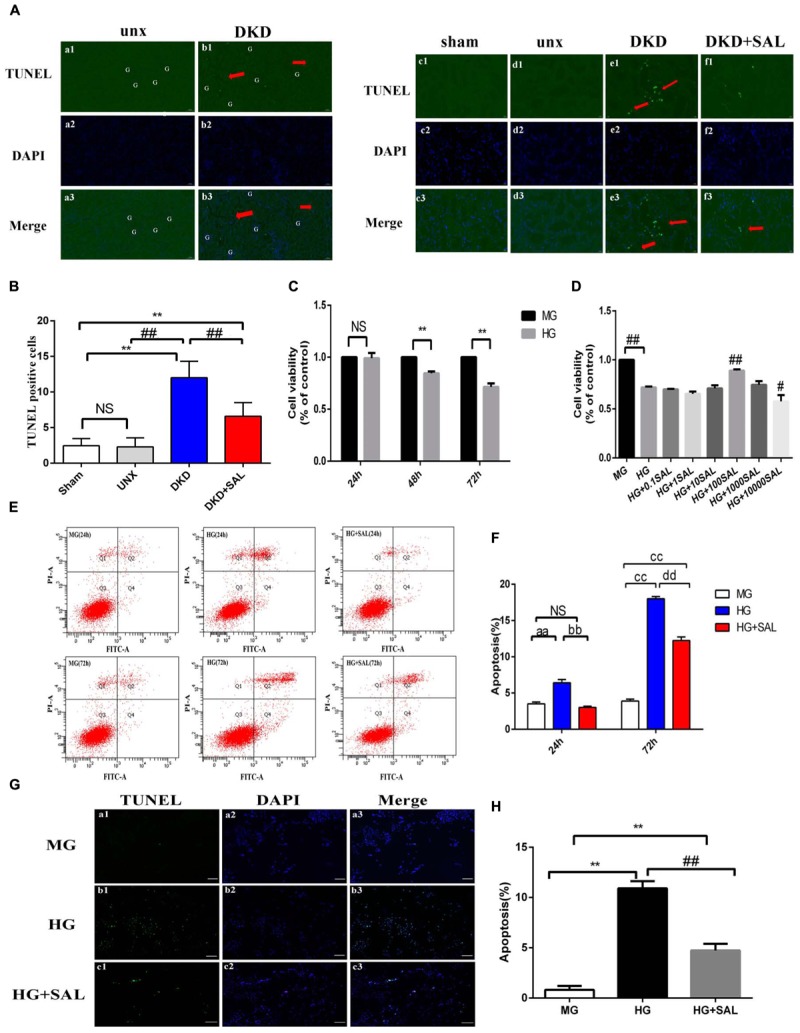
Salidroside inhibited tubular cell apoptosis *in vivo* and *in vitro.* **(A) (a1–a3, b1–b3)** TUNEL-fluorescence was assessed in uninephrectomy (unx) group and diabetes with uninephrectomy (DKD) group, magnification: ×100, scale bar = 100 μm. **(c1–c3, f1–f3)** TUNEL-fluorescence was assessed in sham group, uninephrectomy (unx) group, diabetes with uninephrectomy (DKD) group, and diabetes with uninephrectomy treated with salidroside (DKD + SAL) group, magnification: ×400, scale bar = 20 μm. Arrowheads indicate apoptotic cells in proximal tubules, G, glomerulus. **(B)** Quantitative analysis of TUNEL-positive stained tubular cells in rat kidney cortex. The number of TUNEL-positive cells from 10 random renal cortex tubular microscopic fields (×400) were counted. Data were expressed as positive cell count. Values are expressed as mean ± SE, *n* = (6–8). ^∗∗^*P <* 0.01, vs. sham,^##^*P* < 0.01, vs. DKD. **(C)** Cell viability was assessed by CCK-8 assay in HK-2 cells. Cells were cultured in medium containing 5.5 mM D-glucose + 24.5 mM D-mannitol (MG), 30 mM D-glucose (HG) for 24, 48 or 72 h, respectively. **(D)** Cell viability was assessed by CCK-8 assay in HK-2 cells. Cells were cultured in medium containing 5.5 mM D-glucose + 24.5 mM D-mannitol (MG), 30 mM D-glucose (HG) and 30 mM glucose with salidroside (SAL) at different concentrations (0.1, 1, 10, 100, 1,000, 10,000 μM) for 72 h. Values are expressed as mean ± SE, *n* = 3, ^∗∗^*P* < 0.01, vs. MG, ^#^*P* < 0.05, ^##^*P* < 0.01, vs. HG. **(E,F)** Cells were cultured in medium containing 5.5 mM D-glucose + 24.5 mM D-mannitol (MG), 30 mM D-glucose (HG) and 30 mM glucose + 100 μM SAL for 24 and 72 h. The apoptotic rate of HK-2 cells was stained with Annexin V/PI for flow cytometry analysis. The Q4 (Annexin V-FITC+/PI–) and Q2 (Annexin V-FITC+/PI+) were considered as early stage and late stage of apoptotic cells, respectively. The percent of cell apoptosis was quantified by Q2 + Q4. The results were expressed as the apoptosis rate (%) and shown using a histogram values represent mean ± SE, *n* = 3, ^aa^*P* < 0.01, vs. MG (24 h), ^bb^*P* < 0.01, vs. HG (24 h), ^cc^*P* < 0.01, vs. MG (72 h), ^dd^*P* < 0.01, vs. HG (72 h). **(G,H)** Cells were cultured in medium containing 5.5 mM D-glucose + 24.5 mM D-mannitol (MG), 30 mM D-glucose (HG), and 30 mM glucose + 100 μM SAL for 48 h. Apoptosis was determined by TUNEL staining (green dots) and doubly stained with DAPI (blue dots). The apoptosis rate (%) was calculating by dividing the number of TUNEL positive cells to a population of 100 counted cells per condition, magnification: ×40, scale bar = 200 μm. Values are expressed as mean ± SE, *n* = 3, ^∗∗^*P* < 0.01, vs. MG, ^##^*P* < 0.01, vs. HG.

*In vivo* study, no differences were observed between sham group and unx group regarding tubular cell apoptosis, yet obvious apoptosis was detected in renal tubular cells both in proximal and distal convoluted tubules in the DKD group, especially the proximal tubule (Figure 3A, red arrows). However, the amounts of cells underwent apoptosis in glomerular regions were rare and there were less than three apoptotic cells per 50 glomerulus sections (Figures 3A, a1–b3). We found the apoptotic cells in the DKD groups were higher than those in the unx group (∼10-fold), which were largely inhibited by SAL treatment (approximately 50%, *P* < 0.01, vs. DKD group, Figure 3B).

To further investigate the direct anti-apoptosis effect of SAL on HK-2 cells, we performed cell experiment. Firstly, we examined the cell viability stimulated by HG. As shown in Figure 3C, the cell viability began to decline at 48 h (approximately 20%). After 72 h, there was a more significant decrease (approximately 30%) in viability in medium with 30 mM glucose as compared to the cells incubated in medium with 5.5 mM glucose (*P* < 0.01, Figure 3C). As expected, treatment with SAL (100 μM) significantly increased the cell viability (Figure 3D). Therefore, we selected 100 μM as the concentration of SAL for all subsequent *in vitro* experiments. Secondly, the results of Annexin V-FITC/PI assay showed that there were 3.50% ± 0.44% and 3.90% ± 0.44% apoptotic cells in the MG group and 3.0% ± 0.30% and 12.23% ± 0.87% apoptotic cells in the HG + SAL group, as compared with 6.43% ± 0.76% and 18% ± 0.52% apoptotic cells in the HG group, after 24 h and 72 h treatment, respectively (Figure 3E). The data showed that mannitol had no effect on apoptosis, indicating that the increased apoptosis of HK-2 cell did not result from high osmolarity. And SAL treatment could significantly reduce the apoptosis induced by HG in HK-2 cells (*P* < 0.01, vs. HG 24 h, vs. HG 72 h, respectively, Figure 3F). The results of TUNEL assay further confirmed the anti-apoptosis activity of SAL on HK-2 cells (Figures 3G,H).

### BIM Silencing Inhibited High Glucose-Induced HK-2 Cells Apoptosis

Based on the silico analysis, these apoptosis related genes *(BIM, BAX, CAPS3)* were thought to be the important targets in SAL treatment. BIM, one of the major members of Bcl-2 family, is a pro-apoptotic protein with only one Bcl-2 homology (BH3) domain, and is a pivotal regulator inducing intrinsic apoptosis (Kuwana et al., 2005). BIM protein has three major isoforms, among which BimEL is the main isoform involved in the regulation of apoptosis (Akiyama and Tanaka, 2011). Cumulative evidence suggested that HG can increase BIM expression in multiple tissue cells and mediate cell apoptosis (Doiron et al., 2012; Shin et al., 2014).

**FIGURE 4:**
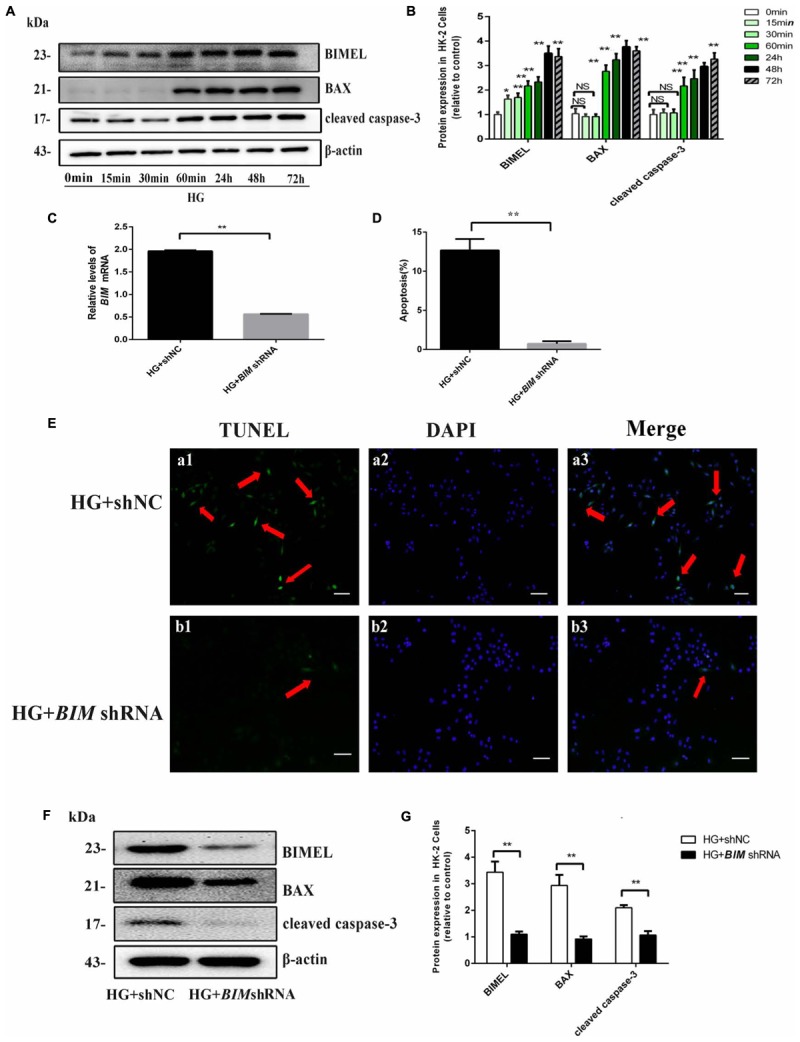
BIM silencing inhibited high glucose-induced HK-2 cells apoptosis. **(A,B)** Cells were cultured in 30 mM D-glucose (HG) medium for 0 min, 15 min, 60 min, 24 h, 48 h, and 72 h, respectively. Western blot and quantitative analysis showed that the expression of BIMEL, BAX, and cleaved caspase-3 protein increased in time-dependent manner, when HK-2 cells were exposed to HG ambience. Values represent mean ± SE, *n* = 3, ^∗^*P* < 0.05, ^∗∗^*P* < 0.01, vs. HG (0 min). **(C)** After transfection with shNC, *BIM* shRNA, HK-2 cells were cultured in an HG medium for 48 h and then harvested for RNA. The mRNA expression of BIM was examined by real time quantitative PCR analysis. **(D,E)** HK-2 cells transfected with shNC, *BIM* shRNA were cultured in an HG medium for 48 h. Apoptosis was determined by TUNEL staining (green dots) and doubly stained with DAPI (blue dots). The apoptosis rate (%) was calculating by dividing the number of TUNEL positive cells to a population of 100 counted cells per condition, and shown using a histogram, magnification: ×100, scale bar = 100 μm. **(F,G)** HK-2 cells transfected with shNC, *BIM* shRNA were cultured in an HG medium for 48 h and then harvested for protein. Western blot and quantitative analysis of BIMEL, BAX, and cleaved caspase-3 and β-actin expression. Values are expressed as mean ± SE, *n* = 3. ^∗∗^*P* < 0.01, vs. HG + shNC.

**FIGURE 5:**
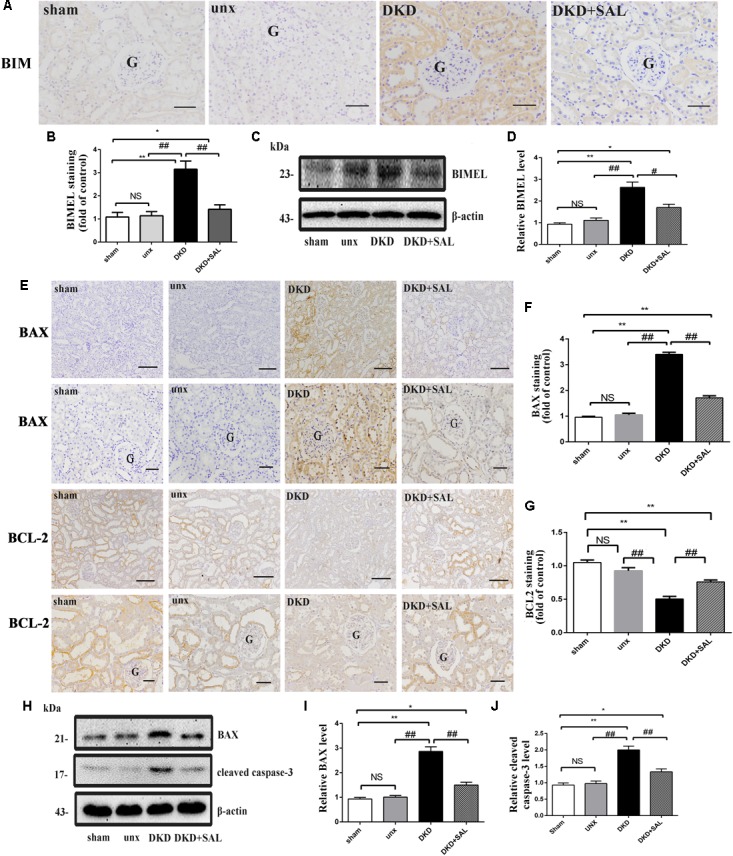
Salidroside suppresses the expression of BIM, BAX, and cleaved caspase-3 in diabetic rats. **(A,B)** Immuno-histochemical studies revealed an increased expression of BIM in the tubular cell in DKD group, while SAL treatment inhibited the increase. BIM existed mainly in cytoplasm of renal tubular epithelial cells, magnification: ×200, scale bar = 50 μm, G, glomerulus. **(C)** Similar expression patterns were seen by western blotting analyses. **(D)** Quantification of average band intensity of western blots. **(E–G)** Immunohistochemical staining and semiquantitative analysis of BAX and BCL2, magnification: ×100 and ×200, scale bar = 150 μm and 50 μm, G, glomerulus, respectively. **(H–J)** Western blot and quantitative analysis revealed the obviously increased expression of BAX and cleaved caspase-3 examined in DKD group, while SAL treatment inhibited the increase. Values are expressed as mean ± SE, *n* = 6–8. ^∗^*P* < 0.05, ^∗∗^*P* < 0.01, vs. sham, ^#^*P* < 0.05, ^##^*P* < 0.01, vs. DKD.

*In vivo* study, we found BIM is mainly expressed in proximal renal tubules and is hardly expressed in the glomeruli (Figure 5A), which were in line with the study of O’Reilly et al. (2000). Our previous study had revealed the obviously up-regulated expression of BIM protein induced by HG in HK-2 cells (Zhang et al., 2017). In the present study, western blotting results showed that HG induced significant time-dependent increases in the expression of BIM, BAX, and cleaved caspase-3 proteins (Figures 4A,B). The results suggested that BIM may play an irreplaceable role in apoptosis of PTECs induced by diabetes.

Our previous study found BIM silence could restrain cytochrome c releasing from mitochondria induced by HG in HK-2 cells (Zhang et al., 2017). To better understand the function of BIM in HG-induced apoptosis, we silenced the endogenous BIM protein by shRNA transfection. As shown in Figures 4C,F, the mRNA and protein expression of BIM both decreased in *BIM* shRNA-transfected cells, suggesting that the transfected *BIM* shRNA effectively inhibited BIM expression. Subsequently, the expression of BAX and cleaved caspase-3 proteins both exhibited reduce in *BIM* shRNA-transfected cells compared with their significant increase in the shNC-transfected cells (both *P* < 0.01 vs. HG + shNC, Figures 4F,G). Furthermore, the result of apoptosis determined by TUNEL revealed that BIM silence completely reversed the apoptosis of HK-2 cells induced by HG (approximately, ∼10-fold). Those results indicated that BIM was a sufficient regulator to switch apoptosis in HK2 cells (Figures 4D,E).

### Salidroside Inhibited the Expression of BIM, BAX, and Cleaved Caspase-3 *in vivo* and *in vitro*

According to Figures 5A,B, an obvious increase expression of BIM protein was detected in DKD group when compared to sham group or unx group by immunohistochemistry (*P* < 0.01), while treatment with SAL markedly decreased the expression of BIM (*P* < 0.01, vs. DKD). The western blot confirmed the finding (*P* < 0.05, vs. DKD, Figures 5C,D). Furthermore, the western blot also showed that the expression of BAX and cleaved caspase-3 proteins were both significantly stronger in the DKD rats compared with that of the non-DKD rats, while treatment with SAL markedly decreased the expression (both *P* < 0.01 vs. DKD group, Figures 5H–J). Subsequently, we detected the expression of BAX and BCL-2 by immunohistochemistry. As illustrated in Figure 5E, treatment with SAL significantly decreased the expression of BAX (*P* < 0.01, vs. DKD) (Figure 5F), while enhanced the expression of BCL2 (*P* < 0.01, vs. DKD) (Figure 5G).

**FIGURE 6:**
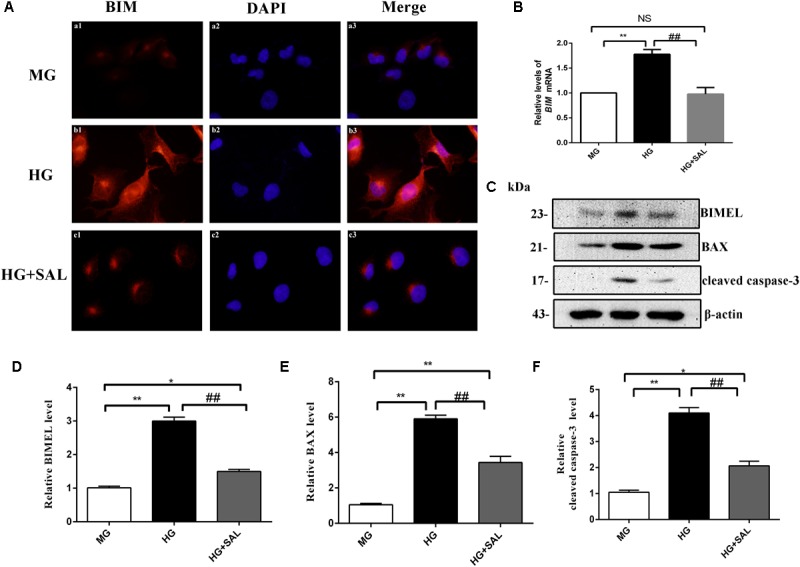
Salidroside suppresses the expression of BIM, BAX, and cleaved caspase-3 in HK-2 cells. **(A)** HK-2 cells were seeded on sterile glass coverslips in a 6-well plate and stimulated with medium containing 5.5 mM D-glucose + 24.5 mM D-mannitol (MG), 30 mM D-glucose (HG) and 30 mM glucose + 100 μM SAL. Two days later, the slides were fixed and the BIM protein was stained and observed under a fluorescence microscope as described in “Materials and Methods” (magnification ×600). **(B–D)** HK-2 cells were exposed to isoosmotic mannitol, or high glucose for 48 h with or without SAL (100 μmol/L) treatment. Then the cells were harvested for RNA and protein. SAL completely inhibited the mRNA and protein expression of BIMEL induced by high glucose in HK-2 cells, assessed using real time quantitative PCR analysis and western blot. **(E,F)** The quantitative analysis of western blot showed that SAL treatment also inhibited the expression of BAX and cleaved caspase-3 induced by high glucose. Values are expressed as mean ± SE, *n* = 3, ^∗^*P* < 0.05, ^∗∗^*P* < 0.01, vs. MG, ^##^*P* < 0.05, vs. HG.

Next, we investigated this effect *in vitro*. The HK-2 cells were exposed to isoosmotic mannitol, or high glucose with or without SAL (100 μM) treatment for 48 h. The location and concentration of BIM were visualized using immunofluorescence microscopic analysis (Figure 6A). As illustrated in Figures 6C–F, western blotting revealed that SAL treatment markedly decreased the expression of BIM, BAX and cleaved caspase-3 proteins induced by HG (both *P* < 0.01 vs. HG). Meanwhile, SAL treatment completely reduced the mRNA level of BIM (*P* < 0.01, vs. HG, Figure 6B). Taken together, these results demonstrate that anti-apoptosis mechanism of SAL induced by HG maybe attribute to the inhibition of BIM-mediated apoptosis pathway.

### Salidroside Inhibited ROS Production of Renal Tubules *in vivo* and *in vitro*

**FIGURE 7:**
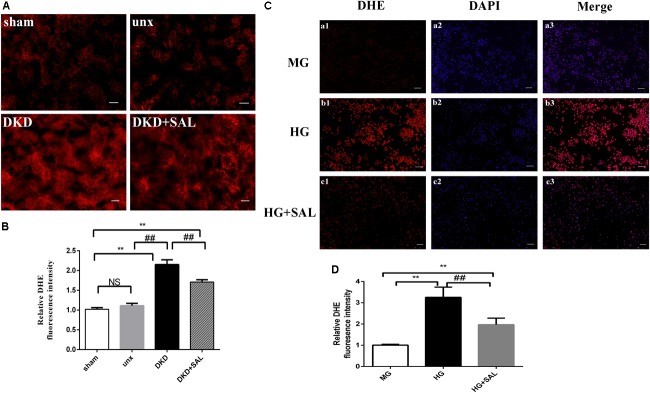
Salidroside decreased ROS production of tubular cell *in vitro* and *in vivo.* **(A)** Representative imaging of DHE fluorescence in kidney (red staining), magnification: ×200, scale bar = 30 μm. **(B)** DHE fluorescence intensity in randomly selected areas of digital images were assayed. Values are expressed as mean ± SE, *n* = (6–8). ^∗∗^*P* < 0.01, vs. sham, ^##^*P* < 0.01, vs. DKD. **(C)** HK-2 cells were exposed to isoosmotic mannitol, or high glucose for 24 h with or without SAL (100 μmol/L) treatment. Detection of ROS in the HK-2 cells. magnification: ×40, scale bar = 150 μm. **(D)** The semiquantitation of DHE fluorescence intensity in HK-2 cells. SAL significantly inhibited the ROS production in HK-2 cells induced by high glucose. Values are expressed as mean ± SE, *n* = 3, ^##^*P* < 0.01, vs. HG.

As shown in Figure 7, increased ROS production was observed both in tubular cells of diabetic rats and HK-2 cells exposure to HG, while SAL significantly prevented the increase. These results indicated that the nephroprotective effect of SAL may be related to oxidative stress inhibition of renal proximal tubular cells. The inhibited effect of SAL on ROS production may participate in regulating the expression of BIM protein.

## Discussion

With the increasing patients and limited therapeutic options, DKD is a growing problem worldwide. As a complementary and alternative medicine, traditional Chinese medicine (TCM) and its main components have been proven to possess satisfactory effectiveness toward DKD (Han et al., 2017; Shao et al., 2017). *Rhodiola rosea* has been reported to have beneficial effects on DKD (Wang et al., 2013). Recent studies also suggested SAL could inhibit high glucose-induced mesangial cell proliferation and extracellular matrix accumulation (Yin et al., 2009; Wang et al., 2017). However, the underlying mechanism of SAL in DKD prevention is not still completely clear. The present study explored the molecular mechanism of SAL in treatment of DKD systematically using the silico analysis. Our experimental study elucidated the renal protective effects of SAL from the perspectives of urinary protein, renal function parameters and histomorphology. Furthermore, the possible nephroprotective mechanisms of SAL might be partially associated with apoptosis suppression of PTECs in response to hyperglycemia.

Considerable evidences suggest that the pivotal locus for the progression of DKD is the tubule. Our study provides evidence that the apoptosis of PTECs in DKD rats is more obvious than that in control rats. Similarly, the apoptosis rate of HK-2 cells is also increased by high-glucose *in vitro*. Both results indicate that the apoptosis of PTECs is an inevitable result of glucotoxicity in the kidney. As tubular epithelial cell loss stimulates remnant tubular hypertrophy. Excessive filtered glucose is reabsorbed by the remaining hypertrophied tubule, leading to further injury of tubules. Some researchers revealed that the apoptosis of PTECs is a crucial contributor to the hyperglycemia-induced kidney failure (Bamri-Ezzine et al., 2003). Inhibiting the apoptosis of PTECs may prevent the vicious cycle of tubular injury and development tubulo-interstitial fibrosis (Docherty et al., 2006).

BIM, a proapoptotic protein with only one BCL-2 homology (BH3) domain, was found to express only in renal tubules. Our results showed that the expression of BIM was markedly up-regulated in DKD rats and HG-induced HK-2 cells. Evidences have suggested that the activated BIM protein could directly or indirectly initiate the activation of BAX. The activated BAX protein could damage the outer mitochondrial membrane and promote mitochondrial membrane permeability. Consequently, the release of cytochrome c from the mitochondria triggers activation of the initiator caspase-3, setting off a chain of events leading to the eventual destruction of the cell (Czabotar et al., 2009). In the present study, silence of BIM protein by *BIM* shRNA significantly relieves the cell apoptosis induced by high glucose. The result indicates that BIM is an important upstream target in high glucose induced apoptosis of PTECs.

SAL significantly inhibited the increased expression of BIM, BAX, and cleaved caspase-3 protein induced by HG, which contribute to the remission of apoptosis in PTECs. Meanwhile, SAL could suppress the transcriptional expression of BIM mRNA, suggesting that some other pathway upstream to the BIM/BAX/CASP3 might be involved in DKD. The mechanism of SAL in inhibiting BIM expression is not clear. In the Figure 2A, the targets associated with *BIM* (gene name, *BLC2L11*) included *ERK2* (gene name, *MAPK1*), *JNK1* (gene name, *MAPK8*) and *AKT1.* Those results suggested that the JNK signaling pathway (Putcha et al., 2003), ERK signaling pathway (Matsumoto et al., 2011) or PI3K/AKT signaling pathway (Zhang et al., 2013) might participate in regulating the transcriptional expression of BIM.

Our results showed SAL alleviated diabetes induced-oxidative stress in tubular cells *in vitro* and *in vivo*. As we know, increased production of ROS sourced from mitochondrion and NADPH oxidase was observed in diabetic kidney tissue (Zhu K. et al., 2015; Schiffer and Friederich-Persson, 2017). The excessive ROS can induce apoptosis of renal tubule cells (Allen et al., 2003). Several lines of evidence have recently shown that ROS-induce apoptosis via activation of extracellular signal-regulated kinase (ERK), which has been shown to transcriptionally increase BIM expression (Glotin et al., 2006; Zhuang et al., 2007). However, active ERK is also known to phosphorylate BimEL, resulting in ubiquitination and degradation of BimEL (Ley et al., 2003). Therefore, ERK activation is expected to reduce the amount of BimEL proteins, leading to cell survival if the proteasome maintains its normal functions. Nevertheless, some researchers have reported that proteasome activity can be reduced when cells were continuously exposed to ROS (Ishihara et al., 2011). SAL, a compound with a phenol glycoside chemical, has significantly antioxidant bioactivity. It has reported that SAL could alleviate high glucose-induced oxidative stress in rat glomerular mesangial cells and alloxan-induced diabetic mice (Li et al., 2011; Wang et al., 2017). Such results indicated that the abnormal accumulation of intracellular ROS might be a joint mechanism, which participated in regulating the transcriptional expression of BIM and protease degradation. The detailed mechanism of SAL in those processes needs further investigation.

Despite these important discoveries, this study has limitations. Firstly, although we found no side effects of SAL in DKD rats, further experimental validation is necessary to confirm the safety of SAL in treatment DKD. Secondly, the other key targets, such as *MMP9, MMP2* (Dang et al., 2008) and *TP53* (Al-Khayal et al., 2016) can induce apoptosis on their own. Thus, continued research is needed to confirm the roles of them in the anti-apoptosis process of SAL. Thirdly, the pathway analysis suggested that SAL may also exert protective action on anti-inflammation and anti-fibrosis. Fortunately, the silico analysis has become an assessment tool to provide comprehensive insights into the underlying mechanisms of drugs and to point out directions for our further research.

## Conclusion

In conclusion, salidroside opens a new window for the treatment of DKD. The nephroprotective effects of salidroside may be partially attributed to the apoptosis inhibition of the proximal renal tubular cells. And the apoptotic protein BIM might be an important target of salidroside in this process.

## Author Contributions

All authors listed have made substantial contributions to the study. CG, YL, and JS performed the experiments. RZ and YZ analyzed the data. JZ and JY took part in the designing of the experiments. LL and JD wrote the manuscript. All authors read and approved the final manuscript.

## Conflict of Interest Statement

The authors declare that the research was conducted in the absence of any commercial or financial relationships that could be construed as a potential conflict of interest.
