# Supplementary material for: Protective Effect of Salidroside Against Diabetic Kidney Disease Through Inhibiting BIM-Mediated Apoptosis of Proximal Renal Tubular Cells in Rats
Source: Front Pharmacol. 2018 Dec 4;9:1433. doi: 10.3389/fphar.2018.01433 (PMC6289038; doi:10.3389/fphar.2018.01433)
Supplement: Supplementary file 1 [file Table_1.doc]

**Protective effect of salidroside against diabetic kidney disease via inhibiting BIM-mediated apoptosis of proximal renal tubular cells in rats**

Congcong Guo***^1,2†^***, Yun Li***^3†^***, Rui Zhang^2^, Yaqin Zhang^2^, Junyu Zhao^2^, Jinming Yao^2^, JieSun^1^, Jianjun Dong^4*^ and Lin Liao^2*^

**Supplementary files 1** , **Websites**

**Websites related to network pharmacology analysis**

(1) DrugBank database (<http://www.drugbank.ca/>);

(2) Online Mendelian Inheritance in Man database (OMIM, <http://www.omim.org/)>;

(3) Genetic Association database (GAD, <http://geneticassociationdb.nih.gov/>);

(4) Therapeutic Target database (TTD, https://db.idrblab.org/ttd/);

(5) Kyoto Encyclopedia of Genes and Genomes Pathway database (KEGG, <http://www.genome.jp/kegg/>);

(6) The Comparative Toxico genomics database (CTD, http://ctdbase.org/),

(7) Herbal Ingredients’ Targets database (HIT, <http://lifecenter.sgst.cn/hit/>);

(8) Swiss Target Prediction (<http://www.swisstargetprediction.ch/>);

(9) STITCH 5.0 (<http://stitch.embl.de/>);

(10) ChemMapper (<http://lilab.ecust.edu.cn/chemmapper/>);

(11) UniProt (http://www.uniprot.org);

(12) the String database(http://string-db.org/).

**Table S1: The 259 genes associated with diabetic nephropathy**.

| **Number** | **Gene name** | **Uniprot ID** | **Protein name** | **Database** |
| --- | --- | --- | --- | --- |
| 1 | AGER | Q15109 | Advanced glycosylation end product-specific receptor | TTD/GAD/CTD |
| 2 | CAT | P04040 | Catalase | CTD/GAD |
| 3 | SGK1 | O00141 | Serine/threonine-protein kinase Sgk1 | OMIM |
| 4 | UNC13B | O14795 | Protein unc-13 homolog B | OMIM |
| 5 | CYP27B1 | O15528 | 25-hydroxyvitamin D-1 alpha hydroxylase, mitochondrial | GAD |
| 6 | PAX4 | O43316 | Paired box protein Pax-4 | OMIM |
| 7 | ACTN4 | O43707 | Alpha-actinin-4 | GAD |
| 8 | ZEB2 | O60315 | Zinc finger E-box-binding homeobox 2 | OMIM |
| 9 | PHLPP1 | O60346 | PH domain leucine-rich repeat-containing protein phosphatase 1 | GAD |
| 10 | NPHS1 | O60500 | Nephrin | GAD |
| 11 | GREM1 | O60565 | Gremlin-1 | GAD |
| 12 | JAK2 | O60674 | Tyrosine-protein kinase JAK2 | TTD |
| 13 | VPS4B | O75351 | Vacuolar protein sorting-associated protein 4B | GAD |
| 14 | SERPINB7 | O75635 | Serpin B7 | OMIM |
| 15 | UGT2B17 | O75795 | UDP-glucuronosyltransferase 2B17 | Drugbank |
| 16 | SCAF4 | O95104 | Splicing factor, arginine/serine-rich 15 | GAD |
| 17 | ABCB11 | O95342 | Bile salt export pump | Drugbank |
| 18 | PIGN | O95427 | GPI ethanolamine phosphate transferase 1 | GAD |
| 19 | ABCA1 | O95477 | ATP-binding cassette sub-family G member 2 | Drugbank |
| 20 | GSR | P00390 | Glutathione reductase, mitochondrial | CTD |
| 21 | SOD1 | P00441 | Superoxide dismutase [Cu-Zn] | GAD/CTD |
| 22 | F2 | P00734 | Prothrombin | OMIM |
| 23 | HP | P00738 | Haptoglobin | GAD |
| 24 | REN | P00797 | Renin | CTD |
| 25 | AGT | P01019 | Angiotensinogen | GAD/CTD |
| 26 | TIMP1 | P01033 | Metalloproteinase inhibitor 1 | CTD |
| 27 | FOS | P01100 | Proto-oncogene c-Fos | CTD |
| 28 | MYC | P01106 | Myc proto-oncogene protein | CTD |
| 29 | PDGFB | P01127 | Platelet-derived growth factor subunit B, PDGF subunit B | CTD |
| 30 | TGFB1 | P01137 | Transforming growth factor beta-1 | TTD/GAD/OMIM/CTD |
| 31 | NPPA | P01160 | Natriuretic peptides A | CTD |
| 32 | NPY | P01303 | Pro-neuropeptide Y | GAD |
| 33 | INS | P01308 | Insulin | GAD/CTD |
| 34 | TNF | P01375 | Tumor necrosis factor | GAD/CTD |
| 35 | IFNG | P01579 | Interferon gamma | CTD |
| 36 | IL1A | P01583 | Interleukin-1 alpha | OMIM |
| 37 | IL1B | P01584 | Interleukin-1 beta | OMIM/GAD/CTD |
| 38 | EPO | P01588 | Erythropoietin | GAD |
| 39 | HLA-DQA1 | P01909 | HLA class II histocompatibility antigen, DQ alpha 1 chain | OMIM/GAD |
| 40 | HLA-DQB1 | P01920 | HLA class II histocompatibility antigen, DQ beta 1 chain | GAD |
| 41 | COL1A1 | P02452 | Collagen alpha-1(I) chain | CTD |
| 42 | COL3A1 | P02461 | Collagen alpha-1(III) chain | CTD |
| 43 | APOE | P02649 | Apolipoprotein E | GAD |
| 44 | APOC1 | P02654 | Apolipoprotein C-I | GAD |
| 45 | APOC3 | P02656 | Apolipoprotein C-III | GAD |
| 46 | MBP | P02686 | Myelin basic protein | GAD |
| 47 | FN1 | P02751 | Fibronectin | CTD |
| 48 | AHSG | P02765 | Alpha-2-HS-glycoprotein | GAD |
| 49 | ALB | P02768 | Serum albumin | Drugbank/CTD |
| **50** | **MMP1** | **P03956** | **Interstitial collagenase** | **TTD** |
| 51 | PROC | P04070 | Vitamin K-dependent protein C | OMIM |
| 52 | SOD2 | P04179 | Superoxide dismutase 2 | KEGG/GAD/OMIM/CTD |
| 53 | TP53 | P04637 | Cellular tumor antigen p53 | GAD/CTD |
| 54 | HSPB1 | P04792 | Heat shock protein beta-1 | GAD |
| 55 | CYBB | P04839 | Cytochrome b-245 heavy chain | CTD |
| 56 | IGF1 | P05019 | Insulin-like growth factor I | CTD |
| 57 | APP | P05067 | Amyloid-beta A4 protein | CTD |
| 58 | ITGB3 | P05106 | Integrin beta-3 | GAD |
| 59 | SERPINB2 | P05120 | Plasminogen activator inhibitor 2 | GAD |
| 60 | SERPINE1 | P05121 | Plasminogen activator inhibitor 1 | GAD/CTD |
| 61 | MPO | P05164 | Myeloperoxidase | CTD |
| 62 | CYP1A2 | P05177 | Cytochrome P450 1A2 | Drugbank |
| 63 | CYP2E1 | P05181 | Cytochrome P450 2E1 | CTD |
| 64 | IL6 | P05231 | Interleukin-6 | GAD/CTD |
| 65 | EDN1 | P05305 | Endothelin-1 | GAD/CTD |
| 66 | ICAM1 | P05362 | Intercellular adhesion molecule 1 | GAD/CTD |
| **67** | **JUN** | **P05412** | **Transcription factor AP-1** | **Drugbank/CTD** |
| 68 | INSR | P06213 | Insulin receptor | CTD |
| **69** | **BCHE** | **P06276** | **Cholinesterase** | **Drugbank** |
| 70 | LPL | P06858 | Lipoprotein lipase | GAD |
| 71 | GPX1 | P07203 | Glutathione peroxidase 1 | CTD |
| 72 | ADRB2 | P07550 | Beta-2 adrenergic receptor | Drugbank |
| 73 | UMOD | P07911 | Uromodulin | GAD |
| 74 | ABCB1 | P08183 | Multidrug resistance protein 1 | Drugbank |
| 75 | NR3C2 | P08235 | Mineralocorticoid receptor | TTD |
| **76** | **MMP2** | **P08253** | **72 kDa type IV collagenase** | **Drugbank/CTD** |
| 77 | ADRB1 | P08588 | Beta-1 adrenergic receptor | Drugbank/GAD |
| 78 | CFH | P08603 | Complement factor H | GAD |
| 79 | CYP3A4 | P08684 | Cytochrome P450 3A4 | Drugbank |
| 80 | IGFBP1 | P08833 | Insulin-like growth factor-binding protein 1 | GAD |
| 81 | IL6R | P08887 | Interleukin-6 receptor subunit alpha | OMIM/GAD |
| 82 | GSTM1 | P09488 | Glutathione S-transferase Mu 1 | GAD |
| 83 | HMOX1 | P09601 | Heme oxygenase 1 | CTD |
| 84 | PARP1 | P09874 | Poly [ADP-ribose] polymerase 1 | CTD |
| 85 | LTA4H | P09960 | Leukotriene A-4 hydrolase | Drugbank |
| 86 | CXCL8 | P10145 | Interleukin-8 | CTD |
| **87** | **BCL2** | **P10417** | **Apoptosis regulator Bcl-2** | **CTD** |
| 88 | SPP1 | P10451 | Osteopontin | CTD |
| 89 | CYP2C8 | P10632 | Cytochrome P450 2C8 | Drugbank |
| 90 | CYP2D6 | P10635 | Cytochrome P450 2D6 | Drugbank |
| 91 | GHR | P10912 | Growth hormone receptor | GAD |
| 92 | LIPC | P11150 | Hepatic triacylglycerol lipase | GAD |
| 93 | SLC2A1 | P11166 | Solute carrier family 2, facilitated glucose transporter member 1 | GAD |
| 94 | G6PD | P11413 | Glucose-6-phosphate 1-dehydrogenase | CTD |
| 95 | VDR | P11473 | Vitamin D3 receptor | GAD |
| 96 | EPO | P11678 | Eosinophil peroxidase | OMIM |
| 97 | CYP2C9 | P11712 | Cytochrome P450 2C9 | Drugbank |
| 98 | BMP2 | P12643 | Bone morphogenetic protein 2 | GAD |
| 99 | ACE | P12821 | Angiotensin-converting enzyme | TTD/GAD/Drugbank/KEGG/CTD |
| 100 | MYH7 | P12883 | Myosin-7 | CTD |
| 101 | CYBA | P13498 | Cytochrome b-245 light chain | CTD |
| 102 | CCL2 | P13500 | C-C motif chemokine 2 | CTD |
| 103 | CCL5 | P13501 | C-C motif chemokine 5 | OMIM/GAD |
| 104 | PRKCSH | P14314 | Glucosidase 2 subunit beta | GAD |
| 105 | NCF1 | P14598 | Neutrophil cytosol factor 1 | CTD |
| 106 | SLC2A4 | P14672 | Solute carrier family 2, facilitated glucose transporter member 4 | CTD |
| 107 | IL1R1 | P14778 | Interleukin-1 receptor type 1 | GAD |
| **108** | **MMP9** | **P14780** | **Matrix metalloproteinase-9** | **Drugbank/CTD** |
| 109 | AKR1B1 | P15121 | Aldose reductase | OMIM/GAD |
| 110 | NQO1 | P15559 | NAD(P)H dehydrogenase [quinone] 1 | CTD |
| 111 | VEGFA | P15692 | vascular endothelial growth factor A | KEGG/GAD/CTD |
| 112 | TIMP2 | P16035 | Metalloproteinase inhibitor 2 | CTD |
| 112 | GNB3 | P16520 | Guanine nucleotide-binding protein G(I)/G(S)/G(T) subunit beta-3 | GAD |
| 114 | UGT2B7 | P16662 | UDP-glucuronosyltransferase 2B7 | Drugbank |
| 115 | CD36 | P16671 | Platelet glycoprotein 4 | CTD |
| 116 | NPPB | P16860 | Natriuretic peptides B | GAD/CTD |
| **117** | **PRKCA** | **P17252** | **Protein kinase C alpha type, PKC-A** | **CTD** |
| **118** | **LGALS3** | **P17931** | **Galectin-3** | **GAD** |
| 119 | IL1RN | P18510 | Interleukin-1 receptor antagonist protein | OMIM/GAD |
| 120 | ADRA2C | P18825 | Alpha-2C adrenergic receptor | TTD |
| 121 | CYP11B2 | P19099 | Cytochrome P450 11B2, mitochondrial | GAD |
| 122 | VCAM1 | P19320 | Vascular cell adhesion protein 1 | CTD |
| 123 | NFKB1 | P19838 | Nuclear factor NF-kappa-B p105 subunit | CTD |
| 124 | CYP3A5 | P20815 | Cytochrome P450 3A5 | Drugbank |
| 125 | HNF1A | P20823 | Hepatocyte nuclear factor 1-alpha | GAD |
| 126 | BMP4 | P21275 | Bone morphogenetic protein 4 | GAD |
| 127 | DRD1 | P21728 | D(1A) dopamine receptor | GAD |
| **128** | **IL10** | **P22301** | **Interleukin-10** | **GAD** |
| 129 | UGT1A1 | P22309 | UDP-glucuronosyltransferase 1-1 | Drugbank |
| 130 | CES1 | P23141 | Liver carboxylesterase 1 | Drugbank |
| **131** | **PTGS1** | **P23219** | **Prostaglandin G/H synthase 1** | **Drugbank** |
| 132 | BMP7 | P23359 | Bone morphogenetic protein 7 | GAD |
| 133 | JAK1 | P23458 | JAK1 | TTD |
| **134** | **SLC6A2** | **P23975** | **Sodium-dependent noradrenaline transporter** | **GAD** |
| 135 | GPT | P24298 | Alanine aminotransferase 1 | CTD |
| 136 | EDNRA | P25101 | Endothelin-1 receptor | TTD |
| 137 | FAS | P25445 | Tumor necrosis factor receptor superfamily member 6 | CTD |
| 138 | NFKBIA | P25963 | NF-kappa-B inhibitor alpha | CTD |
| 139 | MAPK3 | P27361 | Mitogen-activated protein kinase 3, MAP kinase 3 | CTD |
| 140 | HNF1B | P27889 | Hepatocyte nuclear factor 1-beta | OMIM |
| 141 | MAPK1 | P28482 | Mitogen-activated protein kinase 1 | CTD |
| 142 | CTGF | P29279 | Connective tissue growth factor | TTD/GAD/OMIM/CTD |
| 143 | NOS3 | P29474 | Nitric oxide synthase, endothelial | GAD/CTD |
| 144 | BDKRB2 | P30411 | B2 bradykinin receptor | OMIM |
| 145 | AGTR1 | P30556 | Type-1 angiotensin II receptor | Drugbank/GAD/CTD |
| 146 | GSTT1 | P30711 | Glutathione S-transferase theta-1 | GAD |
| **147** | **AKT1** | **P31749** | **RAC-alpha serine/threonine-protein kinase,** | **CTD** |
| 148 | CCR1 | P32246 | C-C chemokine receptor type 1 | GAD |
| 149 | CYP2C19 | P33261 | Cytochrome P450 2C19 | Drugbank |
| 150 | SDC2 | P34741 | Syndecan-2 | GAD |
| 151 | NOS2 | P35228 | Nitric oxide synthase, inducible | CTD |
| **152** | **PTGS2** | **P35354** | **Prostaglandin G/H synthase 2** | **CTD** |
| 153 | UGT1A3 | P35503 | UDP-glucuronosyltransferase 1-3 | Drugbank |
| 154 | IRS1 | P35568 | Insulin receptor substrate 1 | CTD |
| 155 | DDIT3 | P35638 | DNA damage-inducible transcript 3 protein | CTD |
| 156 | TGFBR1 | P36897 | TGF-beta receptor type-1 | GAD |
| 157 | SREBF1 | P36956 | Sterol regulatory element-binding protein 1 | CTD |
| 158 | TGFBR2 | P37173 | TGF-beta receptor type-2 | GAD |
| 159 | PPARG | P37231 | Peroxisome proliferator-activated receptor gamma | Drugbank/GAD/OMIM/CTD |
| 160 | ZEB1 | P37275 | Zinc finger E-box-binding homeobox 1 | OMIM |
| 161 | DDOST | P39656 | Dolichyl-diphosphooligosaccharide--protein glycosyltransferase 48 kDa subunit | GAD |
| 162 | AQP2 | P41181 | Aquaporin-2 | OMIM |
| 163 | CCR2 | P41597 | C-C chemokine receptor type 2 | GAD |
| 164 | MTOR | P42345 | Serine/threonine-protein kinase mTOR | CTD |
| **165** | **CASP3** | **P42574** | **Caspase-3** | **GAD/CTD** |
| 166 | MTHFR | P42898 | Methylenetetrahydrofolate reductase | Drugbank/GAD |
| 167 | DCC | P43146 | Netrin receptor DCC | GAD |
| 168 | MAPK8 | P45983 | Mitogen-activated protein kinase 8, MAP kinase 8 | CTD |
| 169 | MAPK9 | P45984 | Mitogen-activated protein kinase 9 | CTD |
| 170 | SLC15A1 | P46059 | Solute carrier family 15 member 1 | Drugbank |
| 171 | BDKRB1 | P46663 | B1 bradykinin receptor | Drugbank |
| 172 | SLCO1A2 | P46721 | Solute carrier organic anion transporter family member 1A2 | Drugbank |
| 173 | FASLG | P48023 | Tumor necrosis factor ligand superfamily member 6 | CTD |
| 174 | SOX2 | P48431 | Transcription factor SOX-2 | GAD |
| 175 | TRPC1 | P48995 | Short transient receptor potential channel 1 | GAD |
| 176 | CARS | P49589 | Cysteine--tRNA ligase, cytoplasmic | GAD |
| 177 | CEBPA | P49715 | CCAAT/enhancer-binding protein alpha | CTD |
| 178 | GSK3B | P49841 | Glycogen synthase kinase-3 beta | CTD |
| 179 | ENTPD1 | P49961 | Ectonucleoside triphosphate diphosphohydrolase 1 | GAD |
| 180 | CCR5 | P51681 | C-C chemokine receptor type 5 | TTD/OMIM/GAD |
| 181 | CASP9 | P55211 | Caspase-9 | CTD |
| 182 | CDH13 | P55290 | Cadherin-13 | GAD |
| 183 | BACE1 | P56817 | Beta-secretase | TTD |
| 184 | SEC61A1 | P61619 | Protein transport protein Sec61 subunit alpha isoform 1 | OMIM |
| 185 | ACTA2 | P62736 | Actin, aortic smooth muscle | CTD |
| 186 | LRRC7 | P70587 | Leucine-rich repeat-containing protein 7 | GAD |
| 187 | SMAD3 | P84022 | Mothers against decapentaplegic homolog 3 | GAD/CTD |
| 188 | CYCS | P99999 | Cytochrome c | CTD |
| 189 | NOTCH1 | Q01705 | Neurogenic locus notch homolog protein 1 | OMIM |
| 190 | PLAUR | Q03405 | Urokinase plasminogen activator surface receptor | OMIM |
| 191 | RELA | Q04206 | Transcription factor p65 | CTD |
| 192 | GFPT2 | Q06210 | Glutamine--fructose-6-phosphate aminotransferase | OMIM |
| 193 | TJP1 | Q07157 | Tight junction protein ZO-1 | CTD |
| **194** | **BAX** | **Q07812** | **Apoptosis regulator BAX** | **CTD** |
| 195 | BCL2L1 | Q07817 | Bcl-2-like protein | CTD |
| 196 | PPARA | Q07869 | Peroxisome proliferator-activated receptor alpha | CTD |
| 197 | CTBP1 | Q13363 | C-terminal-binding protein 1 | GAD |
| 198 | ROCK1 | Q13464 | Rho-associated protein kinase 1 | TTD |
| 199 | NEUROD1 | Q13562 | Neurogenic differentiation factor 1 | GAD |
| 200 | CASP8 | Q14790 | Caspase-8 | CTD |
| 201 | PON2 | Q15165 | Serum paraoxonase/arylesterase 2 | OMIM |
| 202 | NFKBIB | Q15653 | NF-kappa-B inhibitor beta | CTD |
| 203 | CD226 | Q15762 | CD226 antigen | GAD |
| 204 | ADIPOQ | Q96A54 | Adiponectin receptor protein 1 | GAD/CTD |
| 205 | NFE2L2 | Q16236 | Nuclear factor erythroid 2-related factor 2 | CTD |
| 206 | SLC15A2 | Q16348 | Solute carrier family 15 member 2 | Drugbank |
| 207 | MAPK14 | Q16539 | MAP kinase p38 | TTD |
| **208** | **HIF1A** | **Q16665** | **Hypoxia-inducible factor 1-alpha** | **CTD** |
| 209 | HFE | Q30201 | Hereditary hemochromatosis protein | OMIM/GAD |
| 210 | SLC22A6 | Q4U2R8 | Solute carrier family 22 member 6 | Drugbank |
| 211 | MIA3 | Q5JRA6 | Transport and Golgi organization protein 1 homolog | GAD |
| 212 | LTA | Q5STV3 | LTA (LTA protein) | GAD |
| 213 | SUMO4 | Q6EEV6 | Small ubiquitin-related modifier 4 | GAD |
| 214 | SFRP4 | Q6FHJ7 | Secreted frizzled-related protein 4 | Drugbank |
| 215 | PSRC1 | Q6PGN9 | Proline/serine-rich coiled-coil protein 1 | GAD |
| 216 | APOA5 | Q6Q788 | Apolipoprotein A-V | GAD |
| 217 | MTHFD1L | Q6UB35 | Monofunctional C1-tetrahydrofolate synthase, mitochondrial | GAD |
| 218 | CASP12 | Q6UXS9 | Inactive caspase-12 | GAD |
| 219 | CYP2R1 | Q6VVX0 | Vitamin D 25-hydroxylase | GAD |
| 220 | NPHP3 | Q7Z494 | Nephrocystin-3 | OMIM |
| 221 | ANKS1B | Q7Z6G8 | Ankyrin repeat and sterile alpha motif domain-containing protein 1B | GAD |
| 222 | ALPK2 | Q86TB3 | Alpha-protein kinase 2 | GAD |
| 223 | XYLT1 | Q86Y38 | Xylosyltransferase 1 | GAD |
| 224 | MCF2L2 | Q86YR7 | Probable guanine nucleotide exchange factor MCF2L2 | GAD |
| 225 | PLEKHH2 | Q8IVE3 | Pleckstrin homology domain-containing family H member 2 | OMIM/GAD |
| 226 | ZADH2 | Q8N4Q0 | Prostaglandin reductase 3 | GAD |
| 227 | SLC22A8 | Q8TCC7 | Solute carrier family 22 member 8 | Drugbank |
| 228 | SLC2A12 | Q8TD20 | Solute carrier family 2, facilitated glucose transporter member 12 | OMIM |
| 229 | ELMO1 | Q92556 | Engulfment and cell motility protein 1 | OMIM/GAD |
| 230 | ABCC2 | Q92887 | Canalicular multispecific organic anion transporter 1 | Drugbank/CTD |
| 231 | BAD | Q92934 | Bcl2-associated agonist of cell death | CTD |
| 232 | SIRT1 | Q96EB6 | NAD-dependent protein deacetylase sirtuin-1 | CTD |
| 233 | KIRREL1 | Q96J84 | Kin of IRRE-like protein 1 | GAD |
| 234 | CNDP1 | Q96KN2 | Beta-Ala-His dipeptidase | GAD |
| 235 | CNDP2 | Q96KP4 | Cytosolic non-specific dipeptidase | GAD |
| 236 | NEDD4L | Q96PU5 | E3 ubiquitin-protein ligase NEDD4-like | OMIM/GAD |
| 237 | HMCN1 | Q96RW7 | Hemicentin-1 | GAD |
| 238 | SLC22A12 | Q96S37 | Solute carrier family 22 member 12 | Drugbank |
| 239 | MAP3K5 | Q99683 | Mitogen-activated protein kinase kinase kinase 5 | TTD |
| 240 | ACE2 | Q9BYF1 | Angiotensin-converting enzyme 2 | GAD |
| 241 | SEZ6L | Q9BYH1 | Seizure 6-like protein | GAD |
| 242 | ZNF407 | Q9C0G0 | Zinc finger protein 407 | GAD |
| 243 | HLA-DRB1 | Q9GIY3 | HLA class II histocompatibility antigen, DRB1-14 beta chain | GAD |
| 244 | XYLT2 | Q9H1B5 | Xylosyltransferase 2 | GAD |
| 245 | ADNP | Q9H2P0 | Activity-dependent neuroprotector homeobox protein | GAD |
| 246 | UGT1A10 | Q9HAW8 | UDP-glucuronosyltransferase 1-10 | Drugbank |
| 247 | CDH20 | Q9HBT6 | Cadherin-20 | GAD |
| 248 | NPHS2 | Q9NP85 | Podocin | GAD |
| 249 | SLCO1B3 | Q9NPD5 | Solute carrier organic anion transporter family member 1B3 | Drugbank |
| 250 | NOX4 | Q9NPH5 | NADPH oxidase 4 | CTD |
| 251 | SLC2A9 | Q9NRM0 | Solute carrier family 2, facilitated glucose transporter member 9 | Drugbank |
| 252 | FMN2 | Q9NZ56 | Formin-2 | GAD |
| 253 | MBD2 | Q9UBB5 | Methyl-CpG-binding domain protein 2 | GAD |
| 254 | PPARGC1A | Q9UBK2 | Peroxisome proliferator-activated receptor gamma coactivator 1-alpha | CTD |
| 255 | ERRFI1 | Q9UJM3 | ERBB receptor feedback inhibitor 1 | OMIM |
| 256 | UTS2R | Q9UKP6 | Urotensin II receptor | TTD |
| 257 | ZNF236 | Q9UL36 | Zinc finger protein 236 | GAD |
| 258 | SLC22A7 | Q9Y694 | Solute carrier family 22 member 7 | Drugbank |
| 259 | SLCO1B1 | Q9Y6L6 | Solute carrier organic anion transporter family member 1B1 | Drugbank |

**Note:** The bold fonts represent information of the 15 candidate targets.

**Table S2: The 61** targets of SAL

| **Number** | **Gene name** | **Uniprot ID** | **Protein name** | **Database** |
| --- | --- | --- | --- | --- |
| 1 | SLC5A1 | P13866 | Sodium/glucose cotransporter 1 | Swiss Target Predict |
| 2 | SLC5A2 | P31639 | Sodium/glucose cotransporter 2 | Swiss Target Predict |
| 3 | SLC5A4 | Q9NY91 | Low affinity sodium-glucose cotransporter | Swiss Target Predict |
| 4 | SLC5A10 | A0PJK1 | Sodium/glucose cotransporter 5 (by homology) | Swiss Target Predict |
| 5 | SLC5A3 | P53794 | Sodium/myo-inositol cotransporter (by homology) | Swiss Target Predict |
| 6 | SLC5A9 | Q2M3M2 | Sodium/glucose cotransporter 4 (by homology) | Swiss Target Predict |
| 7 | SLC5A11 | Q8WWX8 | Sodium/myo-inositol cotransporter 2 (by homology) | Swiss Target Predict |
| 8 | TYR | P14679 | Tyrosinase | Swiss Target Predict |
| 9 | TYRP1 | P17643 | 5,6-dihydroxyindole-2-carboxylic acid oxidase (by homology) | Swiss Target Predict |
| 10 | DCT | P40126 | L-dopachrome tautomerase (by homology) | Swiss Target Predict |
| 11 | MBNL1 | Q9NR56 | Muscleblind-like protein 1 | Swiss Target Predict |
| 12 | MBNL2 | Q5VZF2 | Muscleblind-like protein 2 (by homology) | Swiss Target Predict |
| 13 | MBNL3 | Q9NUK0 | Muscleblind-like protein 3 (by homology) | Swiss Target Predict |
| 14 | ADORA1 | P30542 | Adenosine receptor A1 | Swiss Target Predict |
| **15** | **MMP1** | **P03956** | **Interstitial collagenase** | **Swiss Target Predict** |
| **16** | **CASP3** | **P42574** | **Caspase-3** | **STITCH** |
| **17** | **AKT1** | **P31749** | **RAC-alpha serine/threonine-protein kinase** | **STITCH** |
| **18** | **HIF1A** | **Q16665** | **Hypoxia-inducible factor 1-alpha** | **STITCH** |
| **19** | **IL10** | **P22301** | **Interleukin-10** | **STITCH** |
| **20** | **MMP9** | **P14780** | **Matrix metalloproteinase-9** | **HIT** |
| **21** | **PTGS2** | **P35354** | **Prostaglandin G/H synthase 2** | **HIT** |
| **22** | **BCL2** | **P10417** | **Apoptosis regulator Bcl-2** | **HIT** |
| **23** | **BAX** | **Q07812** | **Apoptosis regulator BAX** | **HIT** |
| **24** | **MMP2** | **P08253** | **72 kDa type IV collagenase** | **HIT** |
| 25 | ESR1 | P03372 | Estrogen receptor | HIT |
| 26 | UQCR10 | Q9UDW1 | Cytochrome b-c1 complex subunit 9 | Chemmapper |
| 27 | SDHC | Q99643 | Succinate dehydrogenase cytochrome b560 subunit, mitochondrial | Chemmapper |
| 28 | UQCRQ | O14949 | Cytochrome b-c1 complex subunit 8 | Chemmapper |
| 29 | CYC1 | P08574 | Cytochrome c1, heme protein, mitochondrial | Chemmapper |
| 30 | UQCRH | P07919 | Cytochrome b-c1 complex subunit 6, mitochondrial | Chemmapper |
| 31 | SDHB | P21912 | Succinate dehydrogenase [ubiquinone] iron-sulfur subunit, mitochondrial | Chemmapper |
| 32 | UQCRFS1 | P47985 | Cytochrome b-c1 complex subunit Rieske, mitochondrial | Chemmapper |
| 33 | MT-CYB | P00156 | Cytochrome b | Chemmapper |
| 34 | SDHA | P31040 | Succinate dehydrogenase [ubiquinone] flavoprotein subunit, mitochondrial | Chemmapper |
| 35 | ABO | P16442 | Histo-blood group ABO system transferase | Chemmapper |
| 36 | UQCRC2 | P22695 | Cytochrome b-c1 complex subunit 2, mitochondrial | Chemmapper |
| 37 | UQCRC1 | P31930 | Ubiquinol-cytochrome-c reductase complex core protein 1, mitochondrial | Chemmapper |
| 38 | SDHD | O14521 | Succinate dehydrogenase [ubiquinone] cytochrome b small subunit, mitochondrial | Chemmapper |
| 39 | ETFDH | Q16134 | Electron transfer flavoprotein-ubiquinone oxidoreductase, mitochondrial | Chemmapper |
| 40 | GLT6D1 | Q7Z4J2 | Glycosyltransferase 6 domain-containing protein 1 | Chemmapper |
| 41 | GLTP | Q9NZD2 | Glycolipid transfer protein | Chemmapper |
| 42 | AMY2B | P19961 | Alpha-amylase 2B | Chemmapper |
| 43 | PPARD | Q03181 | Peroxisome proliferator-activated receptor delta | Chemmapper |
| 44 | MBL2 | P11226 | Mannose-binding protein C | Chemmapper |
| **45** | **PTGS1** | **P23219** | **Prostaglandin G/H synthase 1** | **chemmapper** |
| 46 | SELE | P16581 | E-selectin | chemmapper |
| **47** | **SLC6A2** | **P23975** | **Sodium-dependent noradrenaline transporter** | **chemmapper** |
| 48 | CYP19A1 | P11511 | Aromatase | chemmapper |
| **49** | **PRKCA** | **P17252** | **Protein Kinase C, alpha** | **chemmapper** |
| 50 | LGALS9 | O00182 | Galectin-9 | chemmapper |
| **51** | **LGALS3** | **P17931** | **Galectin-3** | **chemmapper** |
| 52 | PYGB | P11216 | Brain glycogen phosphorylase | chemmapper |
| **53** | **BCHE** | **P06276** | **Cholinesterase** | **chemmapper** |
| 54 | ABAT | P80404 | 4-aminobutyrate aminotransferase, mitochondrial | chemmapper |
| 55 | SLC28A3 | Q9HAS3 | Solute carrier family 28 member 3 | chemmapper |
| 56 | PYGM | P11217 | Glycogen phosphorylase, muscle form | chemmapper |
| 57 | NR3C1 | P04150 | Glucocorticoid receptor | chemmapper |
| 58 | LGALS1 | P09382 | GALECTIN-1 | chemmapper |
| 59 | LGALS8 | O00214 | Galectin-8 | chemmapper |
| 60 | FKBP1A | P62942 | Peptidyl-prolyl cis-trans isomerase FKBP1A | chemmapper |
| 61 | RARG | P13631 | Retinoic acid receptor gamma | chemmapper |

**Note:** The bold fonts represent information of the 15 candidate targets.
